# Frequency of MRI Low Signal Intensity in the Buccal Fat of Fetuses and Speculation as to What It May Reflect

**DOI:** 10.3390/children11040463

**Published:** 2024-04-12

**Authors:** Shyam Sunder B. Venkatakrishna, Marcelo S. Takahashi, Juan S. Calle-Toro, Sean Schoeman, Juan Sebastian Martin Saavedra, Dana Alkhulaifat, Suraj D. Serai, Savvas Andronikou

**Affiliations:** 1 Department of Radiology, Children’s Hospital of Philadelphia, Philadelphia, PA 19104, USA; 2 Department of Radiology, University of North Carolina School of Medicine, Chapel Hill, NC 27599, USA; 3 Department of Radiology, University of Texas Health Science Center at San Antonio, San Antonio, TX 78229, USA; 4 Department of Neurology, Massachusetts General Hospital, Boston, MA 02114, USA; 5 Department of Radiology, Perelman School of Medicine, University of Pennsylvania, Philadelphia, PA 19104, USA

**Keywords:** buccal fat pad sign, magnetic resonance imaging, fetus, brown adipose tissue

## Abstract

Purpose: We aimed to characterize the fetal buccal fat pad (BFP) on magnetic resonance imaging (MRI) to determine the frequency and types of sequences on which the BFP demonstrates low signal intensity and determine any possible correlation with timing of the MRI during fetal development. Materials and Methods: A retrospective review of all fetal MR studies was performed, and a pediatric radiologist blinded to the referring and final fetal diagnosis as well as outcome evaluated the included cases. A positive buccal fat pad sign (BFS) was recorded as present if a round, symmetric, and bilateral area was seen in the submalar region of the face with the following signal characteristics: T1 hyperintensity, low signal on echo planar imaging (EPI), low signal on true fast imaging with steady-state free precession (TRUFI), and with restriction on diffusion-weighted imaging (DWI). Results: A total of one hundred sixty-seven (167) fetal MRI studies: one hundred fourteen (114) body (68%) and fifty-three (53) neuro (32%) scans were reviewed during the study period. The BFS was most commonly seen on EPI (63%) and TRUFI (49%) sequences. Substantial agreement between TRUFI and EPI (κ = 0.68; *p* < 0.01); moderate agreement between TRUFI and T1 (κ = 0.53; *p* < 0.01) as well as T1 and EPI (κ = 0.53; *p* < 0.01), and fair agreement between EPI and Diffusion (κ = 0.28; *p* < 0.01) was observed. The median gestational age (GA) was 24 weeks (IQR 22–30 weeks). The fetuses with a positive BFS were significantly older (mean GA of 27 weeks or higher) than those without, for each sequence. Conclusions: The focal low signal in the fetal buccal fat pad, termed the fetal BFS, is a commonly encountered normal finding in the majority of fetal MRI scans on TRUFI and EPI sequences. This finding may be related to the presence and development of brown adipose tissue in the buccal fat pad resulting in T2* effects, but further studies are needed in order to confirm this. Further work can incorporate any of the sensitive sequences demonstrating low signal in brown adipose tissue to map its distribution and development in the fetus and beyond.

## 1. Introduction

The buccal fat pad, or corpus adiposum buccae (Bichat’s fat pad), is a trigone-shaped anatomical structure, situated between the buccinator and masseter muscles, that contributes to the aesthetics of the face, helps in the movement of the muscles of face and it is composed of three lobes: anterior, intermediate, and posterior [1,2,3,4,5]. It has been proposed that in term infants, the buccal fat pad aids in suckling through preventing the indrawing of the cheeks and enhancing intermuscular motion [4]. Fetal magnetic resonance imaging (MRI) is useful for prenatal malformation screening and the contrast of soft tissues along with the field of view is better with fetal MRI than other prenatal screening techniques [6].

The continual advancements in magnetic resonance imaging technology and sequences contribute to the ongoing enhancement of fetal visualization, offering more precise diagnostic capabilities. This progress extends to the improved differentiation and identification of smaller anatomical fetal structures. Given the inherent challenge of imaging a frequently moving fetus during fetal MRI, the utility of fast scanning methods becomes paramount. Single-shot fast spin-echo (SS-FSE) or Half Fourier Acquisition Single Shot Turbo Spin Echo (HASTE) T2-weighted imaging stands out as the primary technique for rapid image acquisition. T1-weighted imaging is employed for the visualization of hemorrhage, fat, and calcification. The balanced steady-state free-precession (SSFP) or true fast imaging with steady-state free precession (TRUFI) proves valuable for depicting the fetal heart and vessels. The application of diffusion-weighted imaging (DWI) serves a dual purpose, detecting cerebral infarction and providing additional functional insights into specific organs. Moreover, at our institute, we adopt an echo planar-based (EPI) acquisition tailored for hemorrhage detection, recognizing that the EPI T2*-weighted sequences are optimal for revealing magnetic susceptibility effects associated with hemorrhage. In the course of our clinical fetal MRI investigations, a discernible circular and symmetric area of low signal within the buccal fat pad (BFP) region was noted in certain fetuses across specific sequences. This study aims to delineate the characteristics of the buccal fat pad on MRI, ascertain the frequency and patterns of sequences where the BFP exhibits low signal intensity, and investigate potential correlations with the timing of MRI sessions during fetal development.

## 2. Materials and Methods

The Institutional Review Board (IRB) approval was obtained, and the informed consent was waived for this Health Insurance Portability and Accountability Act (HIPAA)-compliant study. A retrospective review of all the fetal magnetic resonance studies during a one-year period was performed. The magnetic resonance imaging (MRI) reports indicating the presence of head and neck malformations, incomplete studies, or those that did not have a same-day obstetric ultrasound (US) as the MR imaging were excluded. In addition, the corresponding fetal ultrasound reports were used to exclude any patients with craniofacial abnormalities.

The demographic, clinical, and radiological data were extracted from electronic charts and imaging (US and MR) studies. The age, gestational age, and diagnosis were extracted directly from charts. A pediatric radiologist (M.S.T.) blinded to the referring and final fetal diagnosis as well as the outcome, evaluated the included cases.

**Figure 1:**
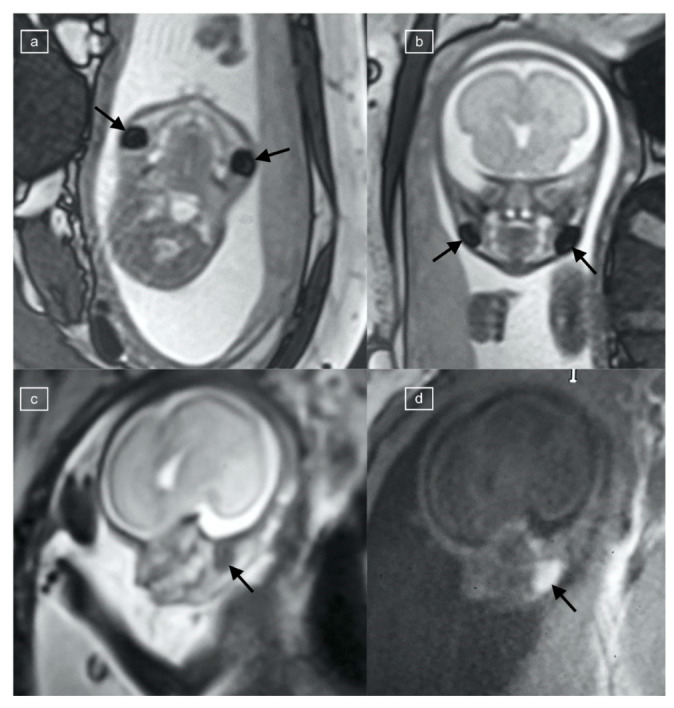
(**a**) Axial, (**b**) coronal, and (**c**) sagittal TRUFI MR images of a 21-week fetus, demonstrating bilateral, oval low-signal-intensity buccal fat pads (black arrows), corresponding to (**d**) T1 VIBE hyperintensity.

**Figure 2:**
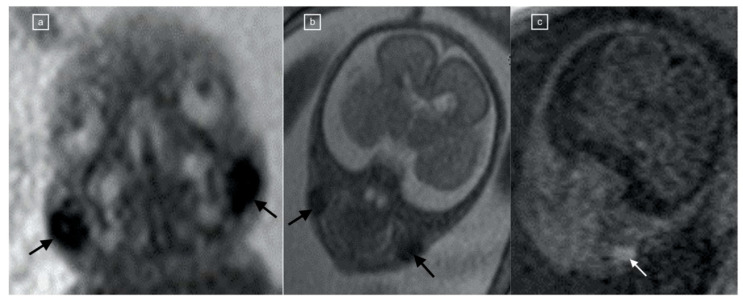
(**a**,**b**) Coronal TRUFI MRI in a 23-week fetus, one of dichorionic diamniotic twins, demonstrating bilateral symmetric, oval low-signal-intensity fat pads (black arrows) corresponding to (**c**) T1 VIBE hyperintensity (white arrow).

**Figure 3:**
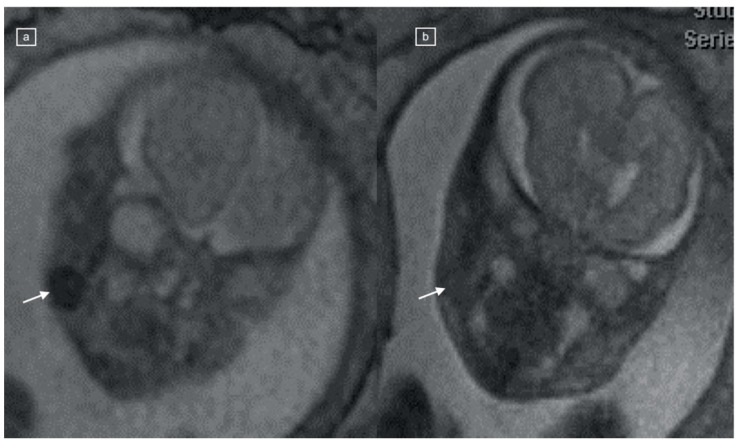
(**a**,**b**) Comparison of TRUFI and HASTE MRI appearances of the fetal buccal fat pad in a 25-week fetus with congenital diaphragmatic hernia. (**a**) Coronal TRUFI demonstrates the oval low-signal-intensity fat pad on the right (arrow), which is isointense to soft tissue on the HASTE image (**b**).

**Figure 4:**
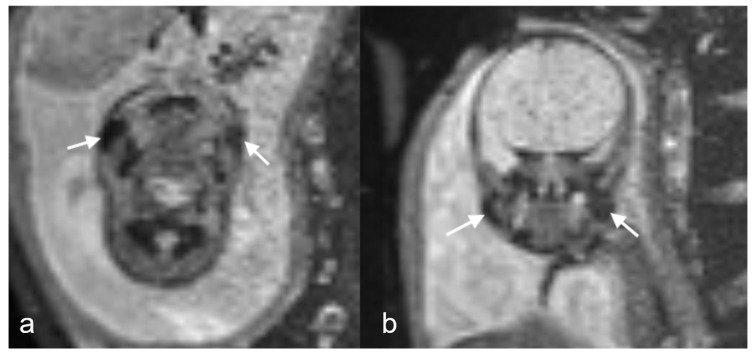
(**a**,**b**) Echo planar imaging at TE42 demonstrates low signal buccal fat pads in this 28-week gestational age fetus with diaphragmatic hernia (arrows).

The sequences reviewed and recorded in this study included: Half Fourier Acquisition Single Shot Turbo Spin Echo (HASTE), true fast imaging with steady-state free precession (TRUFI), T1 weighted imaging (T1), echo-planar imaging (EPI), and diffusion weighted imaging (DWI). The studies were performed on a Siemens 1.5 Tesla scanner, while the 3T studies were obtained on a 3T Skyra or Prisma scanners (Siemens Healthcare, Erlangen, Germany). A positive buccal fat pad sign (BFS) (Figure 1, Figure 2, Figure 3 and Figure 4) was recorded as present if a round, symmetric, and bilateral area was seen in the submalar region of the face with the following signal characteristics: T1 hyperintensity, low signal on EPI, low signal on TRUFI, and with restriction on DWI. The signal on the T2 HASTE was recorded but not used in the definition of the buccal fat pad sign.

### Statistical Analysis

The summary statistics were reported as frequencies and percentages. The comparisons between groups were performed using cross tabulation and the Fisher Exact test. One way analysis of variance (ANOVA) was used to compare the gestational age. A *p*-value < 0.05 was considered statistically significant. The statistical analysis was performed using Statistica™ 14.0.0 (TIBCO Software Inc., Palo Alto, CA, USA).

## 3. Results

A total of one hundred sixty-seven (*n* = 167) fetal MRI studies: one hundred fourteen (n = 114) body (68%) and fifty-three (n = 53) neuro (32%) scans were reviewed during the study period. The most commonly available sequence was HASTE (n = 167), followed by EPI (n = 148), Diffusion (n= 105), T1 (n = 80), and TRUFI (n= 82) (summarized in Table 1). The most common indication for the fetal MR scans overall was ventriculomegaly (n = 50/167, 30%). Most of the studies were performed on a 1.5T magnet strength scanner (n= 152, 91%) while the remaining scans were performed on a 3T scanner (n = 15, 9%), and there was no significant association identified between magnet strength and presence of the positive BFS (*p* > 0.05) for TRUFI, T1, and EPI sequences.

**Figure 5:**
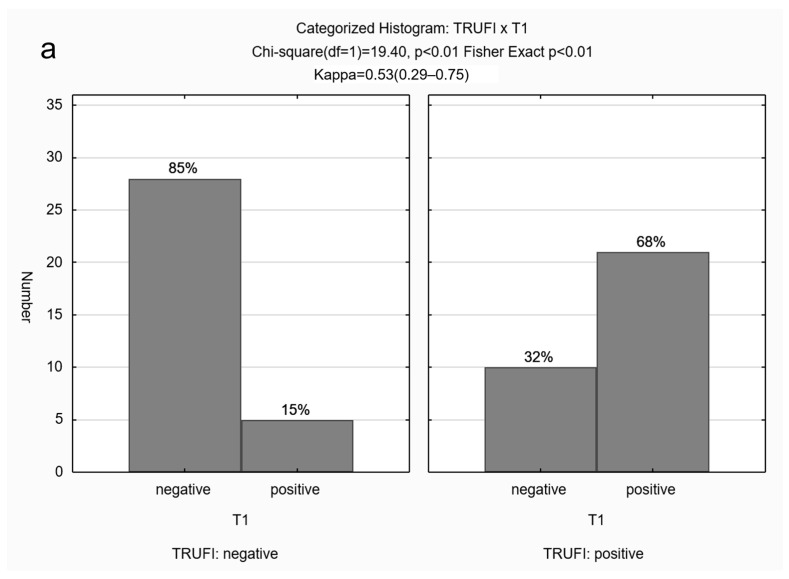

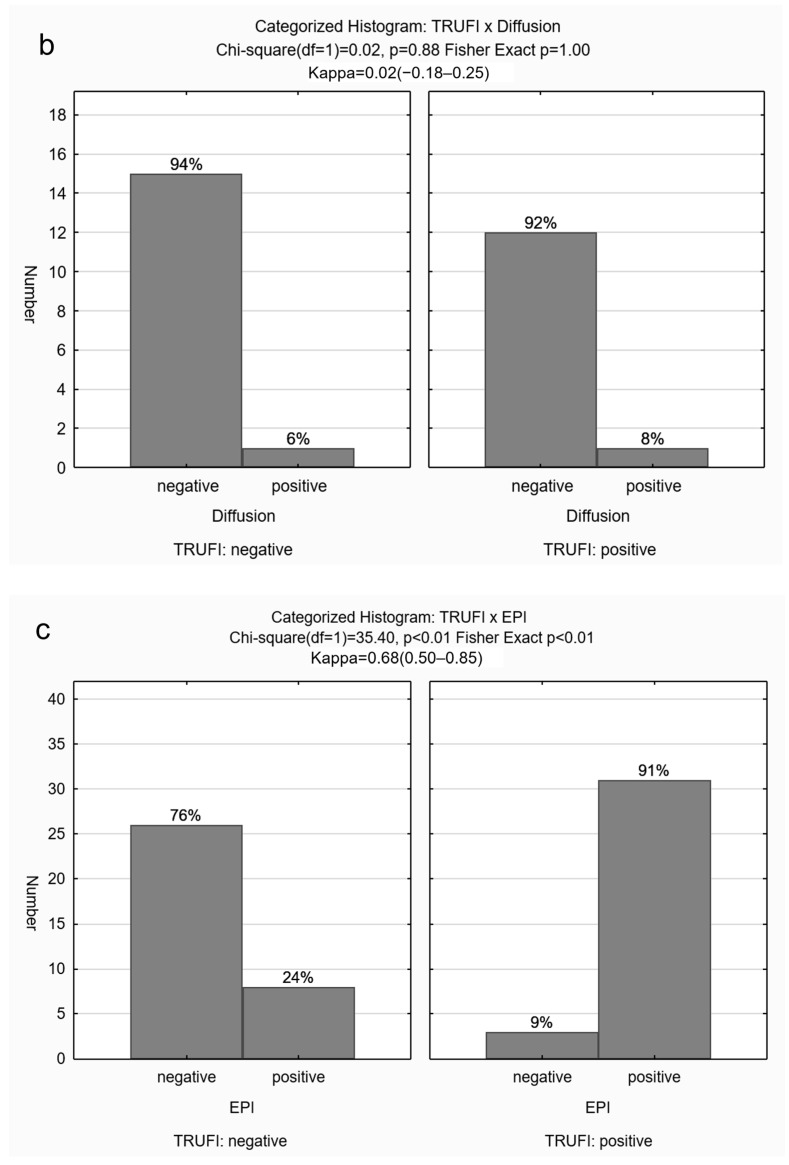

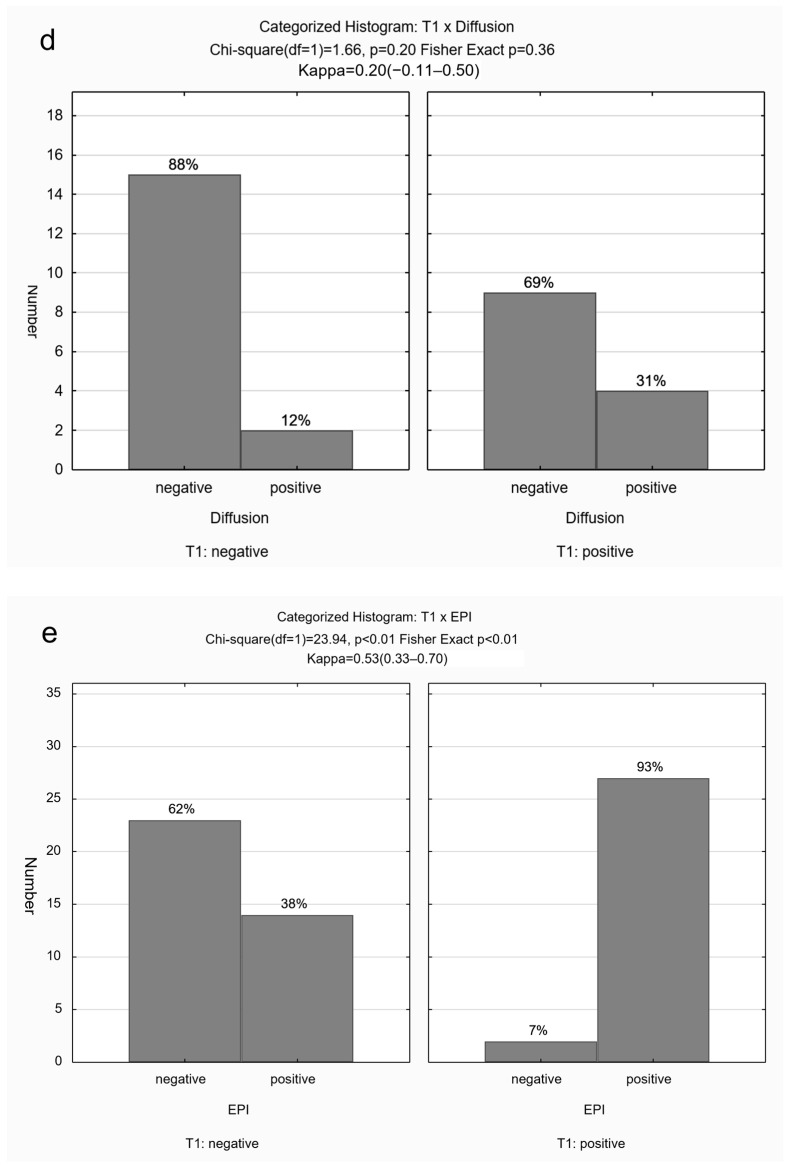

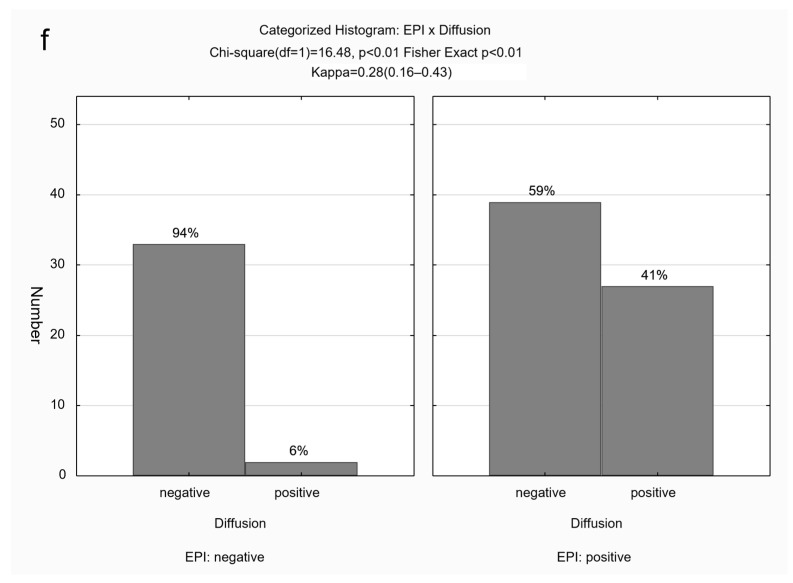
(**a**–**f**) Categorized histograms for demonstrating agreement among the different sequences. TRUFI and EPI (**c**) demonstrated substantial agreement (κ = 0.68; *p* < 0.01); TRUFI and T1 (**a**) and T1 and EPI (**e**) demonstrated moderate agreement (κ = 0.53; *p* < 0.01); EPI and diffusion (**f**) demonstrated fair agreement (κ = 0.28; *p* < 0.01).

The BFS was present in 107/167 (64%) scans on at least one of the sequences: bright on T1, dark on EPI, and dark on TRUFI, with restriction on DWI. It was not visible on HASTE sequences. A positive BFS was observed most frequently on the EPI sequence (Table 1). Concordance among sequences was as follows: Substantial agreement between TRUFI and EPI (κ = 0.68; *p* < 0.01); moderate agreement between TRUFI and T1 (κ = 0.53; *p* < 0.01) as well as T1 and EPI (κ = 0.53; *p* < 0.01), and fair agreement between EPI and diffusion (κ = 0.28; *p* < 0.01) (Figure 5).

**Figure 6:**
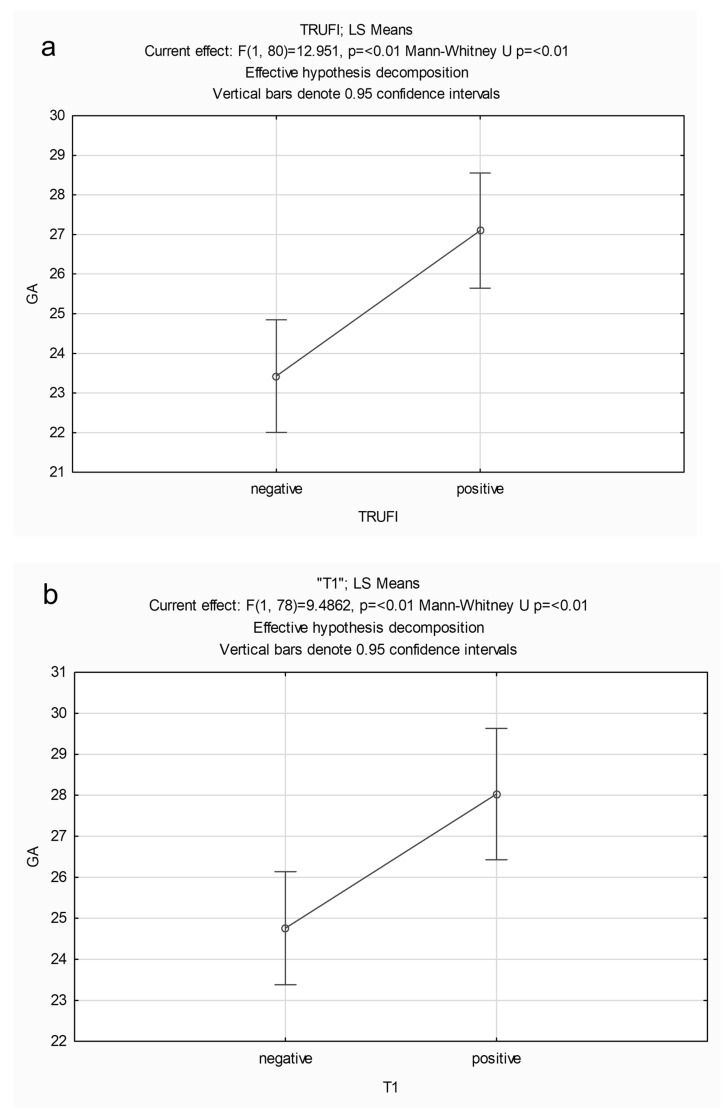

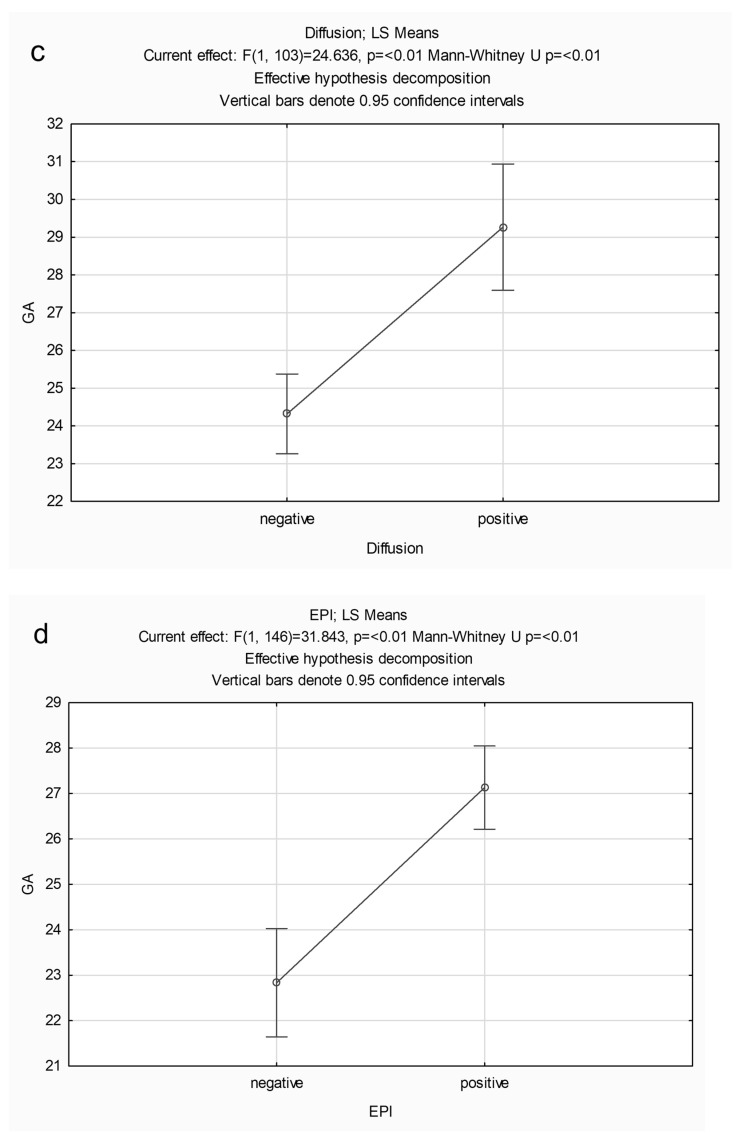
(**a**–**d**) Least squares means (LS Means) of gestational age (GA) for different sequences. The mean GA was 27 weeks or higher for scans with a positive buccal fat pad sign for the different sequences.

The median gestational age was 24 weeks (IQR 22–30 weeks). Based on median age (24 weeks), 82 were above 24 weeks of gestational age (GA) and 85 were 24 or younger. Table 2 provides median and IQR of GA for each sequence and demonstrates that fetuses with a positive BFS were significantly older than those without (mean GA of 27 weeks or higher), for each sequence (Figure 6). The median maternal age was 32.2 years (IQR 28.1–35.9 years) and there was no statistically significant association of maternal age with the positive BFS for any of the sequences (*p* > 0.05).

## 4. Discussion

The buccal fat pad sign (BFS) was present on 64% of fetal MRI scans, on at least one of the sequences: bright on T1, dark on EPI, and dark on TRUFI, with restriction on DWI. In scrutinizing the sequences used for this study, it was determined that echo-planar imaging (EPI) emerged as the most sensitive technique for illustrating the buccal fat pad sign (BFS). Notably, this distinct finding was not perceptible on Half Fourier Acquisition Single Shot Turbo Spin Echo (HASTE) sequences and underscoring the significance of choosing an optimal imaging modality when attempting to characterize the fetal buccal fat pad. The collective observations from this investigation indicate that the signal intensity corresponding to the BFS is a normative occurrence in advanced fetal development, notably manifesting from 27 weeks onwards. Therefore, the likely reasons why there has been lack of recognition of these particular imaging characteristics of the buccal fat pad on MRI are its non-visualization on routine HASTE imaging, which conventionally serves as the primary sequence for brain evaluation, and the timing of most fetal MRI scans for brain assessment, which is before 27 weeks gestation.

A study by Ponrartana et al. describing neonatal buccal fat pad, concluded that it is primarily composed of brown adipose tissue during the first weeks of life, which is replaced by white adipose tissue after that [7]. The presence of brown adipose tissue in the normal fetal buccal fat pad likely explains the signal abnormality seen in some fetal MRI sequences in our cohort and is likely related to late gestational age. The embryologic studies of the buccal fat pad have demonstrated that the adipose tissue differentiates between weeks 14–16 of the second trimester. Generally, from the 23rd week of gestation, the number of fat lobules remain constant, but the growth of the adipose tissue is attributed to an increase in size of this constant number of lobules [8].

Within the human body, two distinct types of adipose tissue exist: white adipose tissue (WAT) and brown adipose tissue (BAT). These adipose tissues serve divergent physiological functions. The white adipose tissue primarily functions as an energy reservoir, characterized by the accumulation of fatty acids and the secretion of hormones. In contrast, the role of brown adipose tissue is intricately linked to the conversion of stored energy into heat. This differentiation in function underscores the complex and dynamic nature of adipose tissues within the broader context of metabolic processes. The interplay between these two adipose tissue types contributes significantly to the regulation of energy homeostasis and thermal balance within the human body [9]. This difference in function is reflected by each tissue’s unique histology. BAT is highly vascularized and rich in brown adipocytes, which have a higher water content and multilocular lipid droplets. Its brownish color is due to its high vascularization and high density of iron-rich mitochondria [10]. This difference in the composition and molecular arrangement of the two types of adipose tissue can be exploited to differentiate BAT from WAT on MR imaging using specific sequences, as has already been demonstrated with chemical shift imaging and MR spectroscopy [11]. Compared to adults, children have a greater prevalence of BAT that is metabolically active and identification of BAT depots requires an understanding of anatomy [12,13].

The distribution of BAT throughout the human body is distinct from WAT, with studies being published intermittently on the subject since 1902 [14,15]. These initial studies were more focused on the neonatal period, and it was then proposed that BAT would gradually disappear or change into WAT with aging. In the last decade, the interest in BAT in humans has emerged again, mainly due to the identification of active BAT in imaging exams of adults and its possible effects on metabolism [16,17,18]. New research has focused on the regulatory role of BAT on the adult body’s energy expenditure, its implication in the obesity epidemic, and its related diseases, and in the development of non-invasive techniques for its identification. Despite this growing interest in BAT, there are very little data published on MRI imaging of fetal BAT distribution [19], quantification, and its clinical relevance in the postnatal period. The BFP sign demonstrated in nearly two thirds of fetal MRI scans likely reflects the presence of BAT.

Our hypothesis gains substantial support from the capability of certain magnetic resonance (MR) sequences to distinguish brown adipose tissue (BAT) from white adipose tissue (WAT) and recent revelations regarding the identification of BAT in the newborn buccal fat pad (BFP) lend further credence to our hypothesis. It is important to note that the histology of BAT affects the associated MRI signal (for different MR sequences). BAT is heavily innervated and vascularized due to its ability to sustain thermogenesis and disperse heat. This creates detectable signal differences in the tissue. Furthermore, BAT has a lower fat-signal fraction than WAT, which is presumed due to its increased metabolic activity. In terms of MRI, this is represented in BAT as a difference in Larmor resonance frequencies. In diffusion-weighted imaging, the metabolically active BAT depletes its intracellular fat stores, thereby water diffusion is less restricted [12].

Attention should also be paid to the relative differences that occur within the domain of BAT. For example, as the fat-signal fraction of BAT is reflective of a “current” state of metabolic activity, the occurrence of metabolically inactive brown adipocytes has been noted in post-mortem studies of neonates [20,21]. Pope et al., in a study of ovine adipose tissue developmental transition, demonstrated four stages of development [22]. This refers to an inherent plasticity in adipose tissue (also demonstrated in rodents) [23] and reflects the metabolic changes that large mammals undergo in the peri-natal period [22,24]. For example, alterations in maternal diet have been demonstrated to affect the growth of different fetal adipose tissue deposits [24].

The buccal fat pad sign (BFS) was defined as a low signal intensity on True Fast Imaging with Steady Precession (TRUFI), a characteristic likely attributed to T2* effects. This distinctive manifestation occurs due to the influence of T2* effects at a steady state, where there is a contribution to the signal from T2*. The extended echo time (TE) values may accentuate local magnetic inhomogeneity, a phenomenon typically employed for detecting features like hemorrhage or calcification. This intricate interplay of magnetic properties adds a layer of complexity to the interpretation of TRUFI signals, necessitating a nuanced understanding of the underlying mechanisms influencing signal intensity in the context of the buccal fat pad [25].

The observed dark signal within the buccal fat on TRUFI could be attributed to the type II chemical shift artifact, a phenomenon typical of gradient echo (GRE) sequences. This artifact occurs when there is an approximate balance between the quantities of fat and water within a pixel, as is the case in metabolically active brown adipose tissue (BAT). The spins of fat and water, having a 180-degree out-of-phase relationship due to their chemical shift or frequency difference, result in signal cancellation. It is noteworthy that the impact of this chemical shift artifact intensifies with higher magnetic field strength, decreased gradient strength, and reduced bandwidth [26].

The presence of low signal on EPI at TE 42 may be due to water diffusion, which is increased in metabolically active BAT. Also, echo-planar imaging is sensitive to T2* decay [25], and this possibly may be the reason for visualization of buccal BAT as low signal.

Regarding the age-based associations found in this retrospective review, it was demonstrated that the mean gestational age for the group with positive BFS was higher (27 weeks or greater) than the group without. This correlates with prior research outlining four developmental phases of adipose tissue in early life, namely: (1) proliferative phase, (2) preparatory phase, (3) thermogenic phase, and (4) lipogenic phase. The proliferative phase occurs in early-mid gestation, which corresponds with our findings of a positive BFS occurring from 27 weeks onwards—i.e., in practice this would not be an expected feature for fetal MRI scans performed between 18–22 weeks gestation but would be a feature for scans performed in late gestation. The physiologic implication is that, as humans are precocial offspring born after a long gestation, with a mature hypothalamic–pituitary–adrenal axis at birth, they are able to switch to non-shivering thermogenesis facilitated by thermogenic BAT, at birth [27,28].

The differential diagnosis of lesions in the lateral face in fetuses and children includes first and second branchial arch cysts (up to 5% of which may be bilateral), dermoid cysts, amygdaloid cysts, and lymphangiomas, especially those involving the parotid gland region. Branchial arch cysts in particular and lymphangiomas may have high protein content internally in the cyst, which may contribute to a high T1 signal but are unlikely to show low signal intensity on true fast imaging with steady-state free precession (TRUFI), a characteristic likely attributed to T2* effects seen with blood products and calcification. Additionally, the differential diagnoses are unlikely to be bilateral. Branchio-oto syndrome (BOS) or branchio-oto-renal syndrome (BORS) with hearing impairment and abnormal phenotype of ears is accompanied by renal malformation and branchial cleft anomalies cysts, which also needs to be considered for bilateral cystic lesions with high T1 signal affecting the face, and evaluation of the ears and kidneys would be useful—none of our patients had features of this syndrome [29].

This study has several implications useful both in clinical practice and for future research. The correlation between positive BFS and older gestational age may be used as an imaging biomarker of normal fetal development or for determining gestational age. Certain fetal MR sequences, such as TRUFI and EPI, are sensitive to T2* effects and can be optimized to identify fetal BAT. This provides the possibility of studying the development of the buccal fat pad. Further study may reveal the relationship of the buccal fat pad to heat and energy metabolism. This may be facilitated through quantitative fat-signal fraction imaging as a biomarker to characterize BAT [12]. The likelihood of brown fat composition of the buccal fat pad through MR imaging may also advise against the recent trends of cosmetic surgery for removing buccal fat pads.

The primary limitation of this investigation lies in its retrospective design. The study comprises MRI scans from a diverse array of MRI scanners from different manufacturers and varying magnetic field strengths, contributing to a heterogeneous collection of analyzed imaging sequences. Given its retrospective nature, facial imaging was not consistently the primary focus. True fast imaging with steady-state free precession (TRUFI), which may form part of routine imaging of the fetal face, was not included in routine brain imaging for an extended period, whereas Half Fourier Acquisition Single Shot Turbo Spin Echo (HASTE) has conventionally served as the staple for fetal brain imaging. Additionally, the absence of post-natal MR imaging in most cases hinders the ability to make comparisons during this period, highlighting a potential avenue for future exploration in a prospective study that contrasts pre-natal and post-natal imaging.

## 5. Conclusions

The observed low signal in the fetal buccal fat, designated as the fetal buccal fat pad sign (BFS) on true fast imaging with steady-state free precession (TRUFI) and echo-planar imaging (EPI) sequences, is a frequently encountered normal feature in the majority of fetal MRI scans. There exists a correlation with gestational age, with higher prevalence manifesting in the later stages of fetal development (beyond 27 weeks of gestation). Our hypothesis suggests a potential association with the presence and maturation of brown adipose tissue in the buccal fat pad, resulting in discernible T2* effects. However, substantiating evidence through further investigations is needed. Radiologists should interpret this finding as a reflection of normal development until comprehensive research is conducted to avert misdiagnosis or unwarranted clinical follow-up. Future studies can leverage sensitive sequences demonstrating low signal in brown adipose tissue (BAT) to delineate its distribution and developmental trajectory in the fetus and beyond.

This may also add utility in further work to quantitatively analyze relative BAT percentages at various gestational ages and examine the impact of maternal diet amongst other entities that affect fetal adipose tissue development, and the potential to prognosticate post-natal outcomes.

## Figures and Tables

**Table 1:** The prevalence of signal abnormality meeting the * Buccal fat pad sign criteria, according to sequence.

Buccal Fat Pad Sign	HASTE		TRUFI		T1		Diffusion		EPI	
	n	%	n	%	n	%	n	%	n	%
Negative	167	100	42	51	46	57	75	71	55	37
Positive	0	0	40	49	34	43	30	29	93	63

* Buccal fat pad sign criteria: T1 hyperintensity, low signal on EPI, low signal on TRUFI, and with restriction on DWI.

**Table 2:** Presence of the buccal fat pad sign (BFS) according to gestational age in weeks.

Buccal Fat Pad Sign	TRUFI (n = 82)		T1 (n = 80)		EPI (n = 148)		Diffusion (n = 105)	
	Median (IQR)		Median (IQR)		Median (IQR)		Median (IQR)	
Negative	21.5	(21, 23)	22	(21, 28)	21	(20, 23)	23	(21, 27)
Positive	27	(23.5, 30)	28	(24, 31)	27	(23, 30)	30	(26, 33)

## Data Availability

The data presented in this study are available on reasonable request from the corresponding author. The data are not publicly available due to privacy restrictions or ethical restrictions.
